# Editorial: Sphingolipid metabolism and cancer

**DOI:** 10.3389/fonc.2022.1049494

**Published:** 2022-11-03

**Authors:** Margarita M. Ivanova, Irina U. Agoulnik, Matilde E. LLeonart

**Affiliations:** ^1^ Department of Translational Research Lysosomal and Rare Disorders Research and Treatment Center, Fairfax, VA, United States; ^2^ Herbert Wertheim College of Medicine, Florida International University, Miami, FL, United States; ^3^ Biomedical Research in Cancer Stem Cells. Vall d’Hebron Research Institute (VHIR), Barcelona, Spain

**Keywords:** sphingolipid, cancer, metabolism, ceramide, anticancer therapy

We are pleased to present this Research Topic entitled “*Sphingolipid Metabolism and Cancer*”, which features review articles focusing on sphingolipid metabolism in the regulation of cancer pathogenesis and therapy.

Bioactive sphingolipids and ceramides modulate the composition of plasma membranes and play a critical role in cell signal transduction (1, 2). Metabolism of sphingolipid is essential for cellular processes, cell growth and proliferation, mitochondrial-autophagy-lysosomal pathways, apoptosis, invasion and metastasis, and immune function (3–5). Moreover, trapping of sphingolipids and their metabolites in lysosomes due to altered sphingolipid metabolism directly affects the lipid compositions of membranes. Accumulating undigested substrates in lysosomes due to deficiencies of various enzymes related to sphingolipid metabolism is linked to more than 50 types of lysosomal storage diseases (6). Recent studies have shown that some sphingolipids and ceramides are involved in cancer progression, malignancy, and drug resistance (7). The knowledge of molecular details of the roles of sphingolipids and their downstream targets in cancer progression opens a new window to develop new therapies to target sphingolipids.

The Research Topic focuses on the functional disruption of sphingolipid metabolism in cancer pathology and a review of novel therapeutic agents targeting sphingolipid metabolism. This Topic gathered a pool of 4 papers, including one research article and three systematic reviews. They all provide essential data on targeting sphingolipids for cancer therapy and cancer drug resistance.

Ceramide synthesis is a central hub of sphingolipid metabolism (8). In addition, ceramide isoforms play a distinct role in the autophagy-lysosomal signaling pathway, ER stress, and apoptosis. C16 ceramide is part of the mitochondrial outer membrane and forms channels that increase permeability and apoptosis (9). Trapika et al. showed the role of C16 ceramide levels in the prostate cancer model. The authors demonstrated that erianin increased the level of C16 ceramide in androgen-dependent prostate cancer cells and triggered cell cycle arrest in castration-resistant cells. Thus, Erianin, a natural compound, shows antitumor effects on both androgen-sensitive and castration-resistant prostate cancer cells. Interestingly, Erianin is a natural bidenzyl composite present in the orchid “*Dendrobium chrysotoxum.”* Authors conclude that Erianin may potentially be a new component of cancer therapies that can target both early and late-stage prostate cancers.

Companioni et al. provide a detailed review regarding targeting sphingolipids as anticancer therapy. Side by side, Figures 2 and 3 demonstrated a comprehensive visual presentation of the sphingolipid metabolic pathway and sphingolipids inhibitors as potential chemotherapeutic agents. The manuscript also included a summary of preclinical and clinical studies of Fenretinide alone and in combination with ABT-199, SAHA, and tamoxifen; Safingol alone and in complex with other drugs, ABC294640, CNL, SKI-II, alpha-GalCer, and Fingolimod.

Li et al. focused their review on new therapeutic targets in sphingolipid metabolism, predictive values, and antitumor immunotherapy resistance. The study explores the function and distribution of sphingolipids and whether this is relevant to cancer progression and diagnosis. They propose different strategies for cancer therapy, such as the use of nanoliposomes to enhance antitumor activity by counteracting the poor solubility and uptake of ceramides. This strategy has been successfully employed to treat Triple-negative breast cancer cells. Finally, the article discusses the remarkable potential of natural compounds based on sphingolipids as antitumor therapies.

Resistance to anticancer drugs continues to be the limiting factor in achieving cures for cancer patients. Review Bataller et al. described the role of sphingolipids metabolism in cancer drug resistance. Altered sphingolipid metabolism has been linked to drug resistance in different types of cancer. This review focuses on the status of enzymes involved in sphingolipid metabolism and metabolites involved in drug resistance. A review of different types of cancer, enzymes involved in sphingolipid metabolism and drug resistance, such as glucosylceramide synthase, sphingosine kinase, acid ceramidase, and sphingomyelinases, is stand out. These enzymes may be considered potential targets for modulating cellular ceramide levels, particularly in treatment-resistant cancers.

Overall, we hope this Research Topic will open new horizons on how to approach and utilize sphingolipid metabolism to combat cancer.

## Author contributions

MI writing, editing, discussion, submission, IA writing, editing, and discussion. ML writing, editing, and discussion. All authors contributed to the article and approved the submitted version.

## Acknowledgments

We are deeply grateful to the Editor-in-Chief, Professor Giuseppe Giaccone, and the editorial board for the opportunity to be the guest editor of this Research Topic.

## Conflict of interest

The authors declare that the research was conducted in the absence of any commercial or financial relationships that could be construed as a potential conflict of interest.

## Publisher’s note

All claims expressed in this article are solely those of the authors and do not necessarily represent those of their affiliated organizations, or those of the publisher, the editors and the reviewers. Any product that may be evaluated in this article, or claim that may be made by its manufacturer, is not guaranteed or endorsed by the publisher.
